# Effectiveness of implant surface debridement using particle beams at differing air pressures

**DOI:** 10.1002/cre2.74

**Published:** 2017-08-02

**Authors:** Max C.T. Wei, Carol Tran, Neil Meredith, Laurence James Walsh

**Affiliations:** ^1^ School of Dentistry The University of Queensland Queensland Australia; ^2^ College of Medicine and Dentistry James Cook University Queensland Australia

**Keywords:** abrasive particle beam, biofilm model, implant surface debridement, surface damage

## Abstract

Because implant surface decontamination is challenging, air powder abrasive systems have been suggested as an alternative debridement method. This in vitro study investigated the effectiveness of different powder formulations and air pressures in cleaning implant surfaces and the extent of surface damage. A validated ink model of implant biofilm was used. Sterile 4.1 × 10 mm Grade 4 titanium implants were coated in a blue indelible ink to form a uniform, visually detectable biofilm‐like layer over the implant threads and mounted into a bone replica material with bony defects to approximate peri‐implantitis. Air powder abrasive treatments were undertaken using glycine, sodium bicarbonate, or calcium carbonate powder at air pressures of 25, 35, 45, and 55 psi. Digital macro photographs of the threads were stitched to give composite images of the threads, so the amount of ink remaining could be quantified as the residual area and expressed as a percentage. Implant surfaces were also examined with scanning electron microscopy to grade the surface changes. No treatment cleaned all the surface of the threads. The powders were ranked in order of decreasing effectiveness and decreasing surface change into the same sequence of calcium carbonate followed by sodium bicarbonate followed by glycine. Higher air pressure improved cleaning and increased surface change, with a plateau effect evident. All powders caused some level of surface alteration, with rounding of surface projections most evident. With air powder abrasive systems, there is a trade‐off between cleaning efficacy and surface damage. Using this laboratory model, sodium bicarbonate and calcium carbonate powders were the most effective for surface cleaning when used at air pressures as low as 25 psi.

## 1 BACKGROUND

The microscopically roughened and hydrophilic surface of titanium fixtures allows rapid attachment and formation of biofilm (Teughels, Van Assche, Sliepen, & Quirynen, 2006). Once the biofilm is established in susceptible patients, destructive inflammatory responses may occur in the surrounding tissue structures, with accompanying soft tissue inflammation and loss of alveolar bone (Zitzmann & Berglundh, 2008). Even though implant‐related diseases such as peri‐implantitis and peri‐implant mucositis have been reported to be relatively common, there is as yet no recognized gold standard approach for the treatment of peri‐implantitis (Esposito, Grusovin, Tzanetea, Piattelli, & Worthington, 2010; Kotsovilis, Karoussis, Trianti, & Fourmousis, 2008; Mahato, Wu, & Wang, 2016).

The ultimate goal of any cleaning process is to decontaminate the surface of the fixture with little alteration (Mann, Parmar, Walmsley, & Lea, 2012; Park, Kim, & Ko, 2012). Modern titanium fixtures have microtextured surfaces created by various combinations of acid‐etching, grit‐blasting, plasma‐spraying, and anodization, to enhance osseointegration (Le Guehennec, Soueidan, Layrolle, & Amouriq, 2007). These microscopic surface irregularities, when combined with protected areas between implant threads, provide physical protection to the biofilm, making professional cleaning with conventional instruments difficult or impossible (Renvert, Samuelsson, Lindahl, & Persson, 2009; Tarafa, Williams, Panvelker, Zhang, & Matthews, 2011).

Various studies have recommended that alternative decontamination methods should be investigated (Armas, Culshaw, & Savarrio, 2013; Mellado‐Valero, Buitrago‐Vera, Sola‐Ruiz, & Ferrer‐Garcia, 2013). Abrasive particle devices have given promising results (Louropoulou, Slot, & Van der Weijden, 2014; Tastepe, van Waas, Liu, & Wismeijer, 2011). Such systems deliver abrasive powder particles where the particles gain their kinetic energy from a stream of water and compressed air (Moene, Decaillet, Andersen, & Mombelli, 2010). Abrasive particles can cause undesirable microscopic alterations of titanium implant surfaces (Tastepe et al., 2011), depending on the nature of the powder used. A recent systematic review supported the use of sodium bicarbonate and glycine powders (Louropoulou et al., 2014).

To date, limited attention has been paid to the selection of particle type and the pressure of compressed air required for effective removal of biofilm (Tastepe et al., 2011). Using a low air pressure should minimize damage to the implant surface and lower the risk of soft tissue injury. The first aim of the present study was to investigate the effectiveness of different powder formulations in removing a biofilm‐like ink from implant surfaces, in terms of cleaning ability and surface damage. The study was conducted under controlled laboratory conditions to remove the influence of confounding factors and clinical variables. The powders tested were those used commonly in the clinical setting, namely, glycine, sodium bicarbonate, and calcium carbonate. The second aim was to explore the influence of air pressure on cleaning ability and surface damage. For this purpose, qualitative analysis from scanning electron microscope images was undertaken, in line with previous assessments of treated implant surfaces (Daood, Bandey, Qasim, Omar, & Khan, 2011; Hallmon, Waldrop, Meffert, & Wade, 1996; Tastepe et al., 2011). The hypotheses tested were that (a) amongst the different powder formulations, calcium carbonate would have the greatest cleaning ability but also impart the greatest change to implant surfaces; and (b) that as air pressure increased, the cleaning ability also increased but surface changes were more pronounced.

## 2 MATERIALS AND METHODS

An in vitro ink model was used (Sahrmann et al., 2015), in which removal of ink on implant surfaces simulates the removal of biofilm. Treated implant surfaces were analyzed using standardized photography and scanning electron microscope.

**Figure 1:**
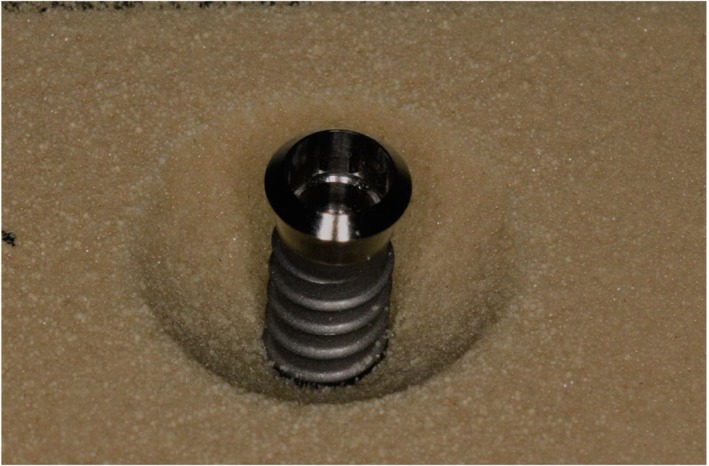
Experimental model showing the defect in Sawbone around the implant fixture

Three sterile 4.1 × 10 mm Grade 4 pure titanium implants (ITC 410, Southern Implants, Irene, South Africa) were coated in blue indelible ink (Sharpie Fine Point Permanent Marker, Sanford L.P., Illinois, USA) to form a uniform, visually detectable biofilm‐like layer over the implant surface, including the valleys between threads (Sahrmann et al., 2015). To verify an even distribution of ink over the surfaces, coated surfaces were inspected under a light microscope at up to 20× magnification. Each implant was mounted in an acrylic resin block (Sawbones, Vashon Island, Washington, USA) that had been prepared with a 6‐mm‐deep defects with a circumscribed saucer‐shaped opening at 60° (Figure 1). to simulate the physical environment of a peri‐implantitis lesion. These defects were the same morphology as the Class Ie defects described by Schwarz et al. (2007). When implants were inserted into the prepared defects, three threads at the coronal region were exposed.

A particle abrasion system (Air‐N‐Go®, Acteon Group, Merignac, France) was used with a subgingival nozzle to treat the surface at compressed air pressures of 25, 35, 45, or 55 pounds per square inch (psi). All powders were sourced commercially (Acteon Group, Merignac, France). Samples in Group A were treated with sodium bicarbonate “Classic” powder (particle size 76 μm), those in Group B with “Perio” glycine powder (particle size 25 μm), and those in Group C with “Pearl” calcium carbonate powder (particle size 55 μm). Each treatment had a duration of 2 min. The nozzle was applied in a freehand manner at a working distance of 1–2 mm with a variable angulation of between 30° and 90° to the implant surface, as recommended by the manufacturer, the same manner as it would be used clinically, attempting to treat all the exposed threads during the two‐min period. Treatments were performed by a single operator (MW), to ensure consistency. There were five replicates for each of the 12 treatment protocols (combinations of differing particle types and air pressures).

**Figure 2:**
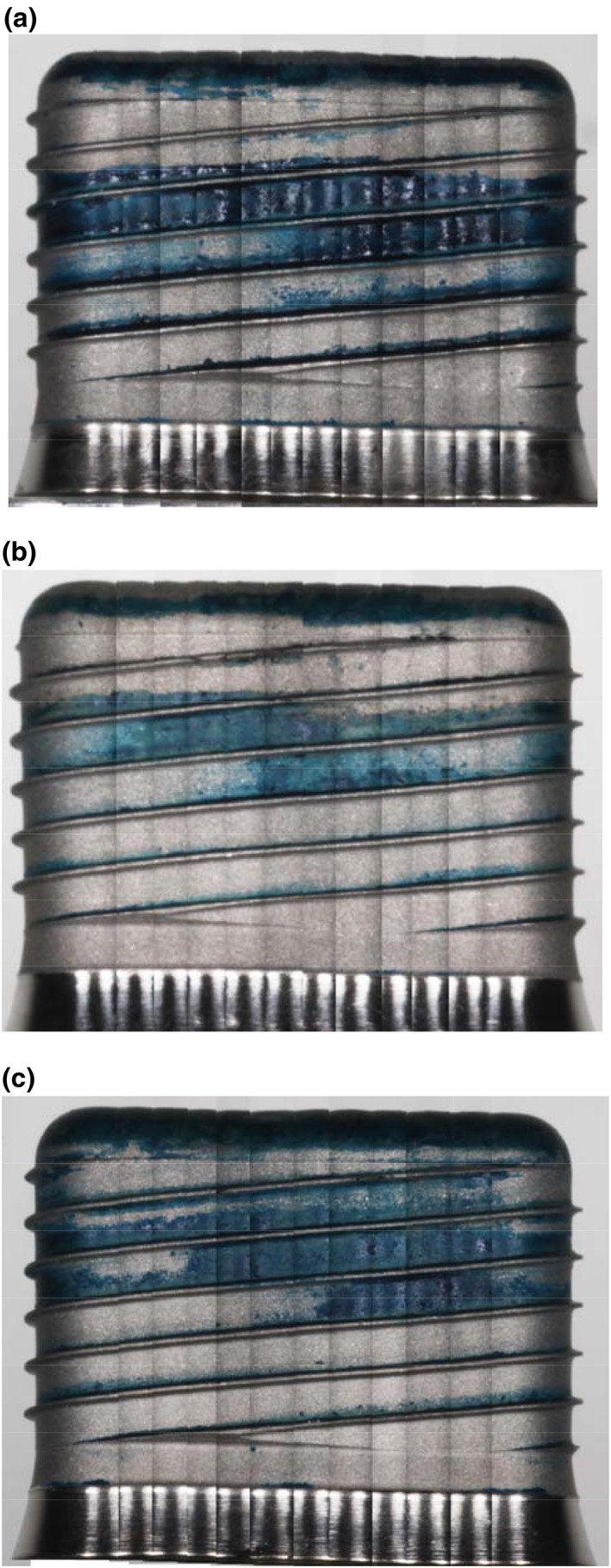
Composite stitched image showing remaining ink after various treatments. The whole surface was covered by ink, but the ink was removed from the most apical section of the implant when it was inserted into the Sawbone and then later removed. Thus, the region of the most apical two threads was excluded from subsequent analysis of surface cleaning effects. Uppermost panel: (a) glycine used at 55 psi, (b) sodium bicarbonate used at 55 psi, and (c) calcium carbonate used at 55 psi

After each treatment, the implant was removed from the Sawbone mount, and any loose powder remnants removed by applying compressed air for 10 s. The implant was then placed on a revolving stand that was marked with 12 even intervals so that 12 photographs of the implant surface could be taken using a digital camera (model 1000D, Canon, Tokyo, Japan) fitted with an 105‐mm macro lens. The images were manually stitched together with Photoshop CC software (Adobe Systems Software, California, USA) to form a rectangular image for analysis (Figure 2). The aperture and shutter speed were set at F32 and 1/4000, respectively.

The whole surface of the fixture was covered by ink, but the ink was removed from the most apical section of the implant when it was inserted into the Sawbone and then later removed. Thus, the region of the most apical two threads was excluded from subsequent analysis of surface cleaning effects.

The composite photographs of the implant surface were analyzed with ImageJ software (Version 1.47, National Institute of Health, Bethesda, USA) to quantify the pixel area of blue ink remaining on the implant surfaces, so that this could be expressed as a percentage of the implant surface area. To compare the results of different treatment protocols, analysis of variance was used (Instat, GraphPad Software, La Jolla, USA). All data sets were checked for normality prior to using parametric tests. The Tukey–Kramer multiple comparison test was used post hoc. The *p* values lower than 0.05 were regarded as significant. After photography, the ink was removed by immersing the implant in three sequential tubes of absolute ethanol for 1 min each, with vigorous agitation at each stage.

One implant was dedicated for each powder type, so that surface damage could be assessed. After the first of the five experimental runs, the implant surface was examined using scanning electron microscopy (Phenom Pro, Phenom‐World BV, Eindhoven, Netherlands). No sputter coating was used. Images were taken at the same locations of the implant (implant collar, before the first, second, and third threads) at 250, 1,000, 2,000, and 5,000× magnification. Damage was scored from images using a qualitative scale, as follows: 0: *no apparent change to the implant surface*; 1: *mild change to the implant surface—slight rounding of surface projections, but no topographical changes*; 2: *moderate change—moderate rounding, with flatter topography*; 3: *moderate change—advanced rounding*; and 4: *pronounced rounding with striations*. Scores were generated by two independent examiners, and the results collated.

## 3 RESULTS

**Figure 3:**
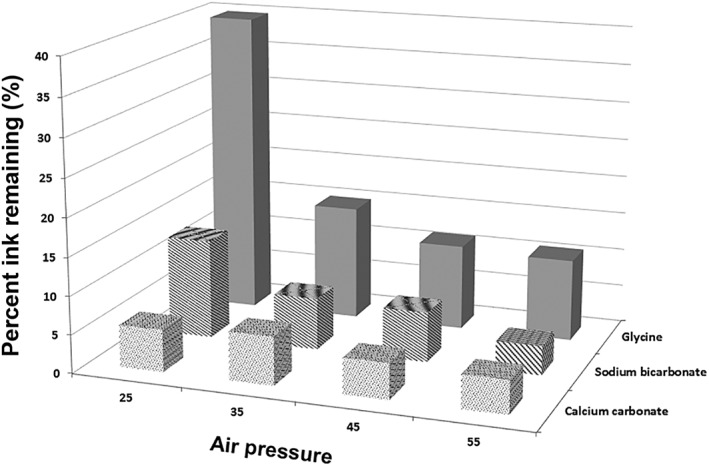
Area of residual ink remaining after using different powder types at varying air pressures. The vertical axis shows remaining ink in percent, thus lower scores indicate better cleaning

The cleaning efficacy of the individual protocols varied according to powder type and air pressure. As summarized in Table 1 and Figure 3, the best cleaning (lowest residual ink area) was seen with calcium carbonate, followed by sodium bicarbonate. Both these powders were more effective than glycine powder. Calcium carbonate reached its maximum cleaning potential at an air pressure of 25 psi (the lowest of all three powders), whereas at air pressures higher than 35 psi, there was no significant difference between calcium carbonate and sodium bicarbonate.

**Table 1:** Implant surface parameters

Powder	Gly	Gly	Gly	Gly	NaB	NaB	NaB	NaB	CaC	CaC	CaC	CaC
Pressure	25	35	45	55	25	35	45	55	25	35	45	55
Area with residual ink (average)	39.74 d	14.98 c	11.21 c	10.72 c	12.98 c	6.76 b	6.52 b	3.80 a	5.50 a	6.30 b	4.68a	4.35 a
SEM score	1	1	1	1	1	1	2	3	2	3	3	4

*Note*. Powder types are designated as the following: Gly = glycine, NaB = sodium bicarbonate, CaC = calcium carbonate. Residual ink area is the mean of five replicates and is expressed as a percentage of the implant surface. Letters indicate groups that are significantly different, from most effective (a) to least effective (d). SEM = scanning electron microscope.

At the lowest air pressure used (25 psi), calcium carbonate gave the best surface cleaning (residual ink area 5.5%), followed by sodium bicarbonate (13.0%), and then glycine (39.7%). Differences between all powder types at 25 psi were statistically significant (*p* < .001). At air pressures of 35, 45, and 55 psi, calcium carbonate and sodium bicarbonate powders continued to be significantly better at cleaning implant surfaces than glycine powder, but results for calcium carbonate and sodium bicarbonate powders were not significantly different despite an overall superior trend for calcium carbonate.

For each particle type, there was an influence of air pressure. For glycine powder used at 25 psi, there was inadequate cleaning with an average of 39.7% of ink remaining. Cleaning performance improved as the air pressure was increased to 35 psi (*p* < .001). Beyond this, the effect showed a plateau, as there was no significant difference between 35 psi and the higher air pressures. Sodium bicarbonate powder used at 25 psi showed a cleaning potential comparable to that of glycine powder used at 55 psi. The cleaning potential increased between 25 and 35 psi and the surface area with ink remaining decreased from an average of 12.98% to 6.76%. Beyond this point, there was a plateau in performance with higher air pressures. In contrast, for calcium carbonate powder, all four air pressures used gave similar results from 25 psi upwards (*p* > .05).

**Figure 4:**
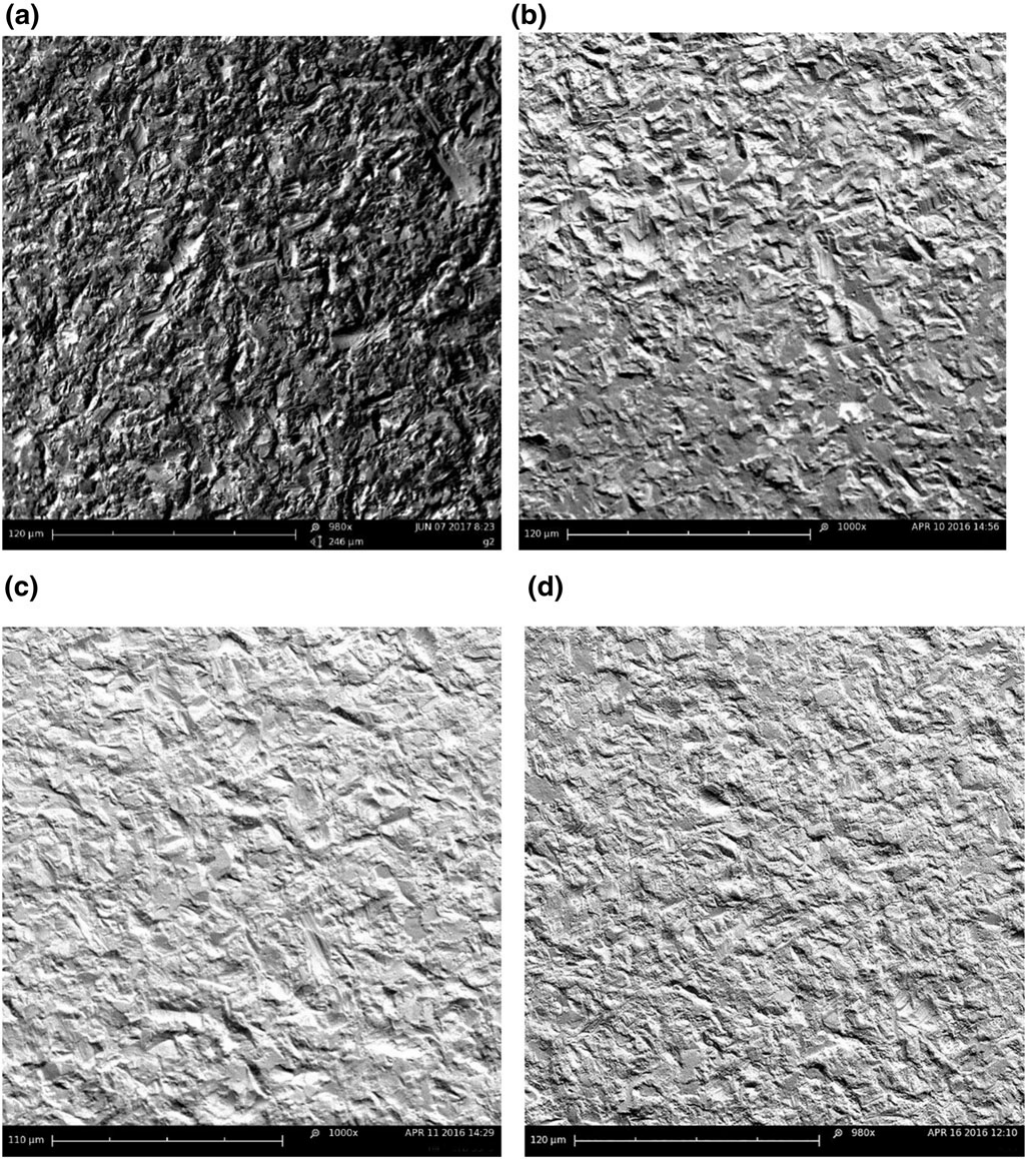
Scanning electron microscope images at 1,000× magnification of treated surfaces. Uppermost left panel: (a) untreated control, (b) glycine used at 55 psi, (c) sodium bicarbonate used at 55 psi, and (d) calcium carbonate used at 55 psi

Scores for surface damage are summarized in Table 1, and representative images of surface effects are shown in Figure 4. Only mild surface alterations were seen with glycine powder, whereas rounding of surface projections occurred with sodium bicarbonate when used at high pressures and moderate surface changes were seen with calcium carbonate at all pressures used, with rounding evident at 25 psi. As air pressure increased, rounding was more pronounced, and striations were noticeable at 55 psi. The changes due to the various treatment protocols were consistent between the various parts of the threads that were imaged. With all powder types, there were areas of the implant surface that could not be accessed regardless of the air pressure used. Areas beneath the threads were consistently found to be the most difficult to access.

## 4 DISCUSSION

This study of the cleaning potential of different powder types at different air pressures shows the influence of both variables, when applied using an ink model to simulate biofilm removal (Sahrmann et al., 2013; Sahrmann et al., 2015). In the present study, a simulated bony defect was used, to replicate the clinical situation where the implant surface is difficult to access (Momber, 2008), providing a realistic challenge for accessing the area of the threads. In previous studies using the ink model, the implant surface was imaged at only one location, whereas in the present investigation, the entire surface was imaged, using the panoramic overview of the entire surface in perfect focus for assessment.

In the present study, glycine not only was found to cause the least surface damage but also was the least effective material for cleaning the surface. Its inferior cleaning potential may reflect it having the lowest density (1.61 g/cm^3^) and smallest particle size (25 μm) of the three materials (Banerjee, Watson, & Kidd, 2000; Momber, 2008; Mount, Walsh, & Brostek, 2005), with a corresponding lower momentum than particles of the other two powder types (Banerjee et al., 2000; Mount et al., 2005) and less energy imparted when it impacts into the surface. The greater hardness of sodium bicarbonate and calcium carbonate (2.5 and 3 respectively on the Mohs hardness scale) compared with glycine (2.0) is also relevant. Better cleaning was found using particles with greater density, namely, sodium bicarbonate and calcium carbonate. These particles were also larger (76 and 55 μm, respectively). A key point of difference between calcium carbonate and sodium bicarbonate is their solubility in water, being low for the former and high for the latter. This parameter could influence the way that particles behave when suspended in a stream of air and water mixed together, as opposed to a stream of compressed air only. The greater amount of undissolved particles of calcium carbonate in a water–air mixture could contribute to enhanced surface abrasion.

For all powder types, when used in an air–water mixture, a plateau in cleaning ability was seen with increasing air pressure, with this effect varying by powder type (glycine and sodium bicarbonate at 35 psi and calcium carbonate at 25 psi). The more positive results seen for sodium bicarbonate and calcium carbonate align with past work using either ink models or in vitro biofilms exposed to particles in an air–water mixture (Augthun, Tinschert, & Huber, 1998; Dennison, Huerzeler, Quinones, & Caffesse, 1994; Parham et al., 1989; Sahrmann et al., 2013; Sahrmann et al., 2015; Schwarz, Ferrari, Popovski, Hartig, & Becker, 2009; Tastepe et al., 2011; Zablotsky, Diedrich, & Meffert, 1992). Both powder types appear suitable for use at lower air pressures than glycine.

Glycine powder caused the least change to the implant surface, which is consistent with the hardness of this material being less than titanium (Cochis et al., 2013; Menini, Piccardo, Baldi, Dellepiane, & Pera, 2015; Tastepe, Liu, Visscher, & Wismeijer, 2013). Changes caused by sodium bicarbonate when used at either low or high air pressures were similar to those described in past studies, with rounding and flattening of the surface topography (Cochis et al., 2013; Menini et al., 2015), even though it is unclear which air pressures had been used in these previous investigations. In the present study, calcium carbonate caused rounding at 25 psi and progressively greater surface changes including striations at 55 psi. How such surface changes influence the biocompatibility of the surface remains to be explored.

An important limitation of the present study was that each implant had progressive treatments, from low to high pressure, meaning that surface damage would accumulate over time. In designing the study, it was recognized that in clinical practice, any one implant surface could be subject to multiple treatments over its service life, so the issue of accumulated surface effects has clinical relevance. The treatments were standardized in all respects so that the relative effects of different powder types could be assessed for the same air pressure.

The present results show that low air pressures (25 psi) appear sufficient for both calcium carbonate and sodium bicarbonate. At low pressures, the chance of damaging soft tissues or causing emphysema is reduced (Armas et al., 2013; Finlayson & Stevens, 1988; McKenzie & Rosenberg, 2009; Moene et al., 2010). Both sodium bicarbonate and calcium carbonate gave promising results when used at low air pressures; however, because of the inherent trade‐off between cleaning and surface damage, further work is needed to optimize particle type and air pressure. This could include the use of profilometry to assess surface changes.
